# Using Nectar-Related Traits to Enhance Crop-Pollinator Interactions

**DOI:** 10.3389/fpls.2018.00812

**Published:** 2018-06-18

**Authors:** Jarrad R. Prasifka, Rachel E. Mallinger, Zoe M. Portlas, Brent S. Hulke, Karen K. Fugate, Travis Paradis, Marshall E. Hampton, Clay J. Carter

**Affiliations:** ^1^Red River Valley Agricultural Research Center, United States Department of Agriculture-Agricultural Research Service, Fargo, ND, United States; ^2^Department of Entomology and Nematology, University of Florida, Gainesville, FL, United States; ^3^Department of Plant and Microbial Biology, University of Minnesota Twin Cities, Saint Paul, MN, United States; ^4^Department of Mathematics and Statistics, University of Minnesota Duluth, Duluth, MN, United States

**Keywords:** nectar, bees, ecosystem services, yield, sunflower, sucrose, breeding, pollination

## Abstract

Floral nectar and other reward facilitate crop pollination, and in so doing, increase the amount and breadth of food available for humans. Though abundance and diversity of pollinators (particularly bees) have declined over the past several decades, a concomitant increase in reliance on pollinators presents a challenge to food production. Development of crop varieties with specific nectar or nectar-related traits to attract and retain pollinating insects is an appealing strategy to help address needs of agriculture and pollinators for several reasons. First, many crops have specific traits which have been identified to enhance crop–pollinator interactions. Also, an improved understanding of mechanisms that govern nectar-related traits suggest simplified phenotyping and breeding are possible. Finally, the use of nectar-related traits to enhance crop pollination should complement other measures promoting pollinators and will not limit options for crop production or require any changes by growers (other than planting varieties that are more attractive or rewarding to pollinators). In this article, we review the rationale for improving crop-pollinator interactions, the effects of specific plant traits on pollinator species, and use cultivated sunflowers as a case study. Recent research in sunflower has (i) associated variation in bee visitation with specific floral traits, (ii) quantified benefits of pollinators to hybrid yields, and (iii) used genetic resources in sunflower and other plants to find markers associated with key floral traits. Forthcoming work to increase pollinator rewards should enable sunflower to act as a model for using nectar-related traits to enhance crop–pollinator interactions.

## Need to Improve Crop–Pollinator Interactions

Production of most world crops depends on bees or other animals to provide or enhance pollination (Klein et al., 2007), an ecosystem service strongly influenced by floral nectar and other rewards. One attempt to assess the value of insect pollination in United States agriculture estimated US$30 billion (Calderone, 2012), while economic valuation of pollination worldwide was valued at €153 billion (Gallai et al., 2009). Though recent estimates of the importance of pollinators in agriculture appear careful and detailed, Melathopoulos et al. (2015) note that pollinator dependence of any single crop is confounded by effects of variety (genotype), environment, and management practices. Nevertheless, without wild and managed bees, various fruit and nut crops would be unavailable and other crops would be less abundant or more costly.

Several distinct trends suggest changes are needed to better manage crop pollination. Honey bee, (*Apis mellifera* L.), the single most significant pollinator worldwide, has suffered substantial declines in colony health and survival in North America and Europe (vanEngelsdorp and Meixner, 2010). Similar negative trajectories have been seen for diversity or abundance of wild bees (Biesmeijer et al., 2006; Potts et al., 2010), which are more important than honey bees for many crops (Garibaldi et al., 2013; Rader et al., 2016). Coincident with pollinator declines, global need for pollinators appears to be increasing, creating a mismatch between pollinator supply and demand (Aizen et al., 2008; Breeze et al., 2014). Following declines in pollinators, price increases in pollinator-dependent crops have been observed (Lautenbach et al., 2012), a trend that likely reflects increased costs of pollination, as per-hive rental fees for honey bees increased more than four fold in just over a decade (Johnson, 2010).

Efforts to address the imbalance of supply and demand for crop pollination logically depend on understanding the problem. Apparent causes for honey bee declines are varied, including diseases and parasites, exposure to pesticides, inadequate nutrition, and increasingly intensive use by humans (United States Department of Agriculture [USDA], 2013; Intergovernmental Science-Policy Platform on Biodiversity and Ecosystem Services [IPBES], 2016). Explanations for negative trends in wild bee abundance or diversity are similar to those for honey bees, but with an emphasis on loss or degradation of non-crop habitats (Klein et al., 2007; Potts et al., 2010). In the United States, efforts to mitigate pollinator declines include improvement in practices related to honey bee health, restoration or enhancement of millions of hectares of land, and restriction or re-evaluation of pesticides (Pollinator Health Task Force, 2015; United States Environmental Protection Agency [US EPA], 2017). England’s national strategy consists of largely voluntary and subsidized measures to support pollinators, including planting wildflowers on farmland and limiting pesticide use through promotion of integrated pest management (Department for Environment Food and Rural Affairs [DEFRA], 2014). One common implication of plans to conserve pollinators and pollination services is the recognition that many different types of measures are needed (Isaacs et al., 2017); with that in mind, crop breeding or selection of varieties that better attract and reward species that pollinate crops appears to be a neglected strategy (but see Palmer et al., 2009; Bailes et al., 2015) that could improve both crop yields and nutritional resources for pollinators.

## Effects of Nectar and Nectar-Related Traits on Crop Pollinators

Nectar is the primary reward for pollinator visitation to wild and cultivated plants, and calories from nectar affect bee growth and development (Burkle and Irwin, 2009). Consequently, variation in nectar has obvious potential to influence pollinator behavior. However, the process of determining which traits are important for crop–pollinator interactions is complicated for several reasons. First, correlations among floral traits are relatively common (Davis, 2000); one trait assessed as influencing behavior in a crop may not be the trait of importance to a pollinator (e.g., flower size versus volume of floral nectar). Second, the state of one trait can easily mask other traits. For example, when floral morphology limits access to nectar (Hawkins, 1969; Erickson, 1975b), nectar quantity or quality are irrelevant to affected pollinators. As a result, nectar and nectar-related traits generally should not be considered to operate independently, but as combinations of reward, cues and other traits which determine plant interactions with pollinators and other insects (Raguso, 2004). Lastly, it is worthwhile to note that the effects of plant traits vary among pollinators; differences in life-histories (social versus solitary) or the identity of a single key pollinator species may determine the effect of nectar and nectar-related crop traits (Tepedino and Parker, 1982).

With the caveats regarding the complexity of crop–pollinator relationships in mind, examples of specific nectar-related traits associated with pollinator activity are noted under subheadings below. The types of traits discussed are well-established as influencing pollinator behavior in non-crop species, and (interspecific) variation in nectar-related traits of non-crop species is the basis for successful habitat manipulations to increase presence or activity of crop pollinators (Campbell et al., 2012; Feltham et al., 2015). Because our emphasis is on cultivated plants, many seminal publications on plant-pollinator interactions are not included. Also, the references are not an exhaustive list, but emphasize crops which show at least a modest increase in production through pollinator activity (see Klein et al., 2007) and studies that link intraspecific variation in nectar-related traits to a pollinator response.

### Nectar Quantity and Quality

Intraspecific variation in the calories available to pollinators from nectar-feeding often helps explain pollinator preferences within a crop, and may arise from differences in nectar volume per flower, concentration of nectar sugars, density of flowers or the duration of flowering. Many fruit and vegetable crops with strong dependence on pollinators, including blueberry (Jabłoński et al., 1984), watermelon (Wolf et al., 1999), raspberries and blackberries (Schmidt et al., 2015), and zucchini (Roldán-Serrano and Guerra-Sanz, 2005) show positive associations between bee visits and nectar volume or total sugar per flower (nectar volume × concentration). Pollination benefits to *Citrus* species and cultivars vary, but nectar volume is correlated with honey bee visitation (and also flower size; Albrigo et al., 2012). Peppers and onions are both considered unattractive to bees and receive little direct benefit from pollinator visitation; however, bees are needed to produce hybrid seed, and increased honey bee visits are associated with increased nectar sugar or volume (Rabinowitch et al., 1993; Silva and Dean, 2000).

Unlike the aforementioned specialty crops, the enormous scale on which soybean is grown provides a significant opportunity to improve crop-pollinator interactions; though this legume is considered self-pollinated, some soybean varieties benefit from pollinator visitation (Erickson, 1975a) and show substantial variation in nectar volume (Erickson, 1975b; Severson and Erickson, 1984). Soybean nectar and bee visits appear positively correlated (Erickson, 1975b; Robacker et al., 1983), but Palmer et al. (2009) suggest more work is needed to directly associate soybean floral traits with pollinator behavior. A similar situation exists for oilseed rape, which varies in nectar volume (Pierre et al., 1999; Bertazzini and Forlani, 2016), an attribute that increases the duration of bumblebee visits to flowers (Creswell, 1999).

Observations of bees foraging on sugar solutions (Waller, 1972; Mommaerts et al., 2013) and nectars from many plant taxa (Baker and Baker, 1983) suggest the ratio of common nectar sugars (sucrose, fructose, and glucose) may influence pollinator choice. Because sucrose is a disaccharide made of glucose and fructose, nectar sugar composition is often shown as a ratio of sucrose to fructose and glucose or as percent sucrose. Sucrose-richness of nectar in crops has only been implicated in pollinator choice for a few crops, including zucchini (Roldán-Serrano and Guerra-Sanz, 2005) and sunflower (Pham-Delègue et al., 1994). However, other crops including oilseed rape (Kevan et al., 1991) and peppers (Roldán-Serrano and Guerra-Sanz, 2004) have been shown to provide nectars that vary from no sucrose to sucrose-rich.

In addition to sugars, nectar contains a wide variety of other components at lower concentrations, including inorganic ions, amino acids, lipids, and secondary plant compounds (see Roy et al., 2017), many of which are attractant or repellent to pollinators. Few studies are available that examine intraspecific variation in these components, and even fewer which link the variation in non-sugar components of nectar to crop pollination. One interesting exception is caffeine; at levels found in coffee and citrus nectars, caffeine improves honey bee memory of a reward (nectar) and its associated cue (odor), suggesting caffeine encourages bees to make repeat visits to flowers of both plant genera (Wright et al., 2013). The amino acid proline, a floral nectar component, seems to increase honey bee preference at concentrations of 2–6 mM (Carter et al., 2006). Though oilseed rape shows significant variation in proline concentration (Bertazzini and Forlani, 2016), its levels may be below the 2 mM threshold to affect pollinator preference. On the other hand, accessions from a soybean wild relative suggest *Glycine* spp. may have proline levels high enough to influence bee foraging (Carter et al., 2006).

In addition to floral nectar, many cultivated plants have extrafloral nectaries. In general, extrafloral nectar is an inducible, indirect defense against herbivores that functions by attracting predators and parasitoids to damaged plants (Heil, 2015). Though extrafloral nectar has little apparent application for enhancing crop–pollinator interactions, it shares much of the quantitative and qualitative diversity found in floral nectar (González-Teuber and Heil, 2009). Because extrafloral nectaries benefit plants by reducing herbivory, Heil (2015) and Stenberg et al. (2015) suggest this indirect defense should be used in breeding crops.

### Other Nectar-Related Traits

Floral scent and appearance also influence pollinator choice. Though there are innate pollinator preferences (Reverté et al., 2016), it is clear that bees use visual and olfactory information as indicators of floral reward, often learning to associate cues and reward. In wild *Brassica rapa*, the amount of the floral volatile phenylacetaldehyde was correlated with floral reward (sugar and pollen per flower), and bumble bees learned a positive response to the volatile after foraging on plants (Knauer and Schiestl, 2015). Preference of a wild bee for strawberry varieties was associated with higher levels of floral volatiles, but correlation of volatiles with reward was not tested (Klatt et al., 2013). Appearance of flowers is important for pollination of apple cultivars; when white-flowered apples were planted with several crabapples as pollen donors, honey bees showed a strong preference for white crabapples (Mayer et al., 1989), possibly due to flower constancy (a pollinator habit of repeatedly visiting one type of flower; Waser, 1986). Honey bees appear to evaluate alfalfa at a distance, as floral display size of individual plants positively influenced honey bee visitation (Bauer et al., 2017). Wild *Brassica rapa* and oilseed rape vary for the presence of nectar guides, an ultraviolet floral pattern visible to bees, which increase pollinator visits to plants (Brock et al., 2016). However, in one comparison, a mutation that causes complete loss of petals in oilseed rape did not appear to reduce honey bee visitation (Pierre et al., 1996).

Aside from providing visual cues, aspects of floral morphology can be important in limiting access to floral reward. The size of opening to access nectar (“throat diameter”) was positively associated with honey bee visitation to highbush blueberry (Courcelles et al., 2013). Floral morphology in some soybean varieties strongly discourages pollinators by production of closed (cleistogamous) flowers; however, because flower type can be controlled by both genotype and environment, bee visitation to some varieties may occur in periodic pulses that coincide with production of open (chasmogamous) flowers (Erickson, 1975b).

Pollen is also a significant floral reward that shows intraspecific variation. The clearest instances where pollen appears to influence pollinator behavior are in male-sterile lines, which may receive more or less bee visits, depending on bee species or nutritional status (Tepedino and Parker, 1982; Soto et al., 2013). Few data are available to generalize how moderate quantitative differences in pollen (e.g., 25–35%; Vear et al., 1990) affect crop–pollination. A succinct summary of nectar-related crop traits and their effects on bees is shown in **Table 1**.

**Table 1:** Nectar-related traits and pollinator responses for selected crops and crop wild relatives.

Species (common name)	Plant trait	Response	Reference
*Allium cepa* (onion)	Nectar volume	+ honey bee visits	Silva and Dean, 2000
*Brassica napus* (oilseed rape)	Nectar volume^∗^	+ bumble bee visits	Creswell, 1999
*Brassica napus* (oilseed rape)	Absence of petals	= /+ honey bee visits	Pierre et al., 1996
*Brassica rapa* (field mustard)	Ultraviolet patterning	+ pollinator visits	Brock et al., 2016
*Brassica rapa* (field mustard)	Floral volatiles	+ bumble bee visits	Knauer and Schiestl, 2015
*Capsicum annuum* (pepper)	Nectar volume × concentration	+ honey bee visits	Rabinowitch et al., 1993
*Citrus* spp. (citrus)	Nectar volume, flower size	+ honey bee visits	Albrigo et al., 2012
*Cucurbita pepo* (zucchini)	Nectar volume, sugar ratios	+ bumble bee visits	Roldán-Serrano and Guerra-Sanz, 2005
*Citrullus lanatus* (watermelon)	Nectar concentration	+ honey bee visits	Wolf et al., 1999
*Fragaria* x *ananassa* (strawberry)	Floral volatiles	+ solitary bee visits	Klatt et al., 2013
*Glycine max* (soybean)	Flower access (cleistogamy)	- honey bee visits	Erickson, 1975b
*Helianthus annuus* (sunflower)	Nectar volume × concentration	+ social bee visits	Tepedino and Parker, 1982
*Helianthus annuus* (sunflower)	Nectar volume, flower size (depth)	+/- pollinator visits	Mallinger and Prasifka, 2017a
*Helianthus annuus* (sunflower)	Flower size (depth)	- wild bee visits	Portlas et al., 2018
*Malus* spp. (apple and crabapple)	Flower color	+ honey bees visits	Mayer et al., 1989
*Medicago sativa* (alfalfa)	Size of floral display	+ honey bee visits	Bauer et al., 2017
*Rubus* spp. (caneberries)	Nectar volume	+ social bee visits	Schmidt et al., 2015
*Vaccinium corymbosum* (blueberry)	Nectar volume × concentration	+ honey bee visits	Jabłoński et al., 1984
*Vaccinium corymbosum* (blueberry)	Flower size (diameter)	+ honey bee visits	Courcelles et al., 2013

*^∗^Surrogate nectar solution dispensed in a range of volumes after removal of naturally secreted nectar. Natural variation in nectar volume for B. napus shown by Pierre et al. (1999) and Bertazzini and Forlani (2016).*

## Improving Sunflower Crop Yields and Resources for Bees

Sunflowers are attractive to both managed and wild pollinators (United States Department of Agriculture [USDA], 2015), but because of selection for self-fertility, are sometimes considered to have a low-to-moderate dependence on bees (Delaplane and Mayer, 2000; Klein et al., 2007). However, for production of hybrid seed, where pollen must be moved between male-fertile and male-sterile lines, bees are critically important (Greenleaf and Kremen, 2006), and otherwise desirable inbred lines are sometimes discarded because of their failure to attract pollinators. Further, although commercial sunflower hybrids may be capable of self-pollination, yields are generally improved by bees (DeGrandi-Hoffman and Chambers, 2006). Traits associated with pollinator attraction and the pollinator-dependence of sunflower hybrids have been previously investigated (Tepedino and Parker, 1982; Sammataro et al., 1983; Pham-Delègue et al., 1994; Dag et al., 2002), but these studies often included few plant genotypes, were published outside of peer-reviewed literature, or used open-pollinated varieties developed without hybrid breeding. As a result, a series of studies has been undertaken by USDA-ARS researchers and collaborators to (i) associate variation in pollinator visitation with specific floral traits, (ii) assess benefits of pollinators to yields of modern sunflower hybrids, and (iii) use genetic resources in sunflower and other plants to facilitate improved sunflower-pollinator interactions. A summary of recently published and new data related to these objectives is provided below.

Field trials in 2014–2015 (Mallinger and Prasifka, 2017a) were designed to associate wild and managed bee visitation to floral traits of inbred lines. For pairs (*n* = 10) of sunflower isolines with or without cytoplasmic male sterility (cms), honey bees favored the pollen-free cms lines while wild bees preferred the male-fertile equivalents. After accounting for the effect of pollen, nectar sugar (volume × concentration) was positively associated with visits by both honey bees and wild bees. Additionally, inbred lines with shorter corollas (=easier access to nectar) were found to receive more pollinator visits. Subsequent work in 2016–2017 (Portlas et al., 2018) focused on the effect of floret size because deeper corollas prevent nectar sampling by short-tongued bees, and because phenotyping floret size should be more rapid and precise than assessing nectar volume. Evaluation of 100 female lines showed total floret length ranged from 6.8 to 9.9 mm. When a subset of these lines was grown again and bee visits counted daily, most of the variation in wild bee counts was explained by floret size. Data from Portlas et al. (2018) suggest that for lines with the longest florets, a reduction in floret size of only 1.0 mm should double bee visitation; further reductions in floret size beyond 1.0 mm provide even greater benefits, likely because proboscis (“tongue”) lengths vary both within (Waddington and Herbst, 1987) and between bee species (Cariveau et al., 2016).

Over the same period, we evaluated pollinator contributions to sunflower yields. Because pollinator benefits to yields of oilseed hybrids were assessed somewhat recently (DeGrandi-Hoffman and Chambers, 2006), we focused on confection sunflowers (i.e., non-oil hybrids used as a snack food or as a food ingredient). Over 2 years in North Dakota, 15 commercial hybrids were grown with or without pollinators excluded (via fine mesh bags). Though some hybrids received no benefit from pollinators, open-pollination by insects increased yields by 26% when averaged across all hybrids, and five of the hybrids showed increases of 39–108% (Mallinger and Prasifka, 2017b). In part, variation in benefits from pollinators was explained by how attractive each hybrid was to bees, though nectar-related traits were not directly assessed for these hybrids. After repeating this work in additional states (South Dakota and Nebraska), early results indicate the effect of pollinators on yields may be greatly influenced by growing conditions. In 2016, yields from 10 tested hybrids saw a <20% increase from open pollination in North Dakota, a benefit of ≈30% in South Dakota and >100% increase over pollinator exclusion in Nebraska (Mallinger, unpublished data). Data from 2017 showed less variation across environments (pollinator benefit of 30–35%), but cumulative results indicate that bees provide a substantial benefit to confection sunflower yields, and that even hybrids that effectively self-pollinate in one location or year may need bees to achieve consistent, high yields.

**FIGURE 1:**
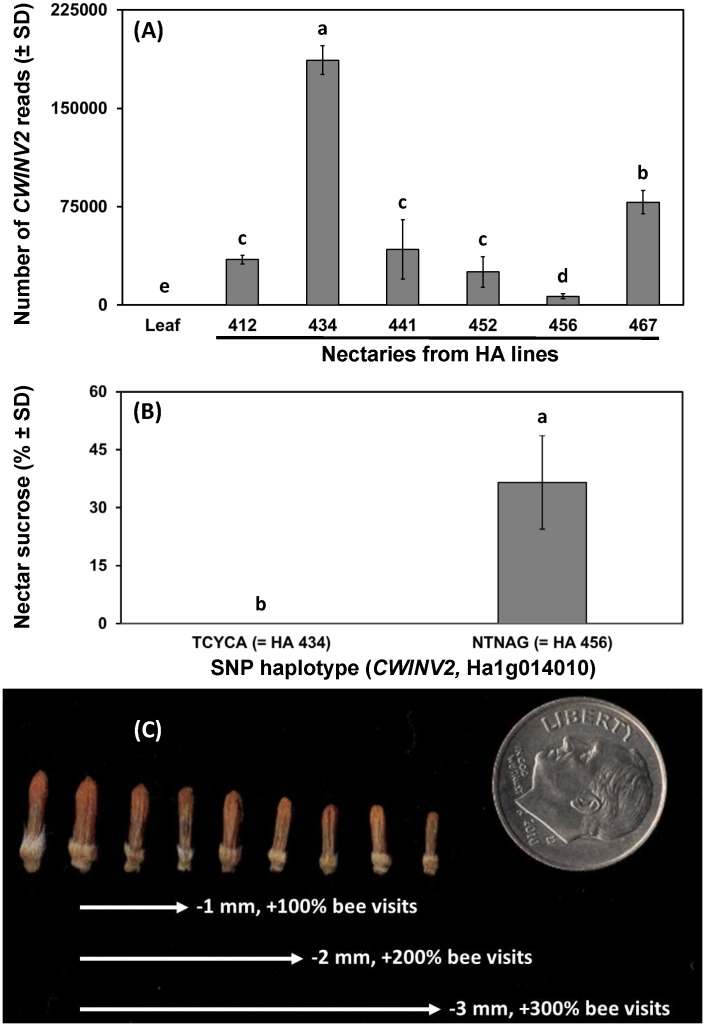
Relationships between nectar-related traits in sunflower and genetic markers or pollinator behavior. Panels indicate **(A)** expression of cell wall invertase (*CWINV2*) from control tissue (leaf) and nectaries of *Helianthus annuus* maintainer (HA) lines previously phenotyped for sucrose content, **(B)** sucrose content (% by mass of sucrose + fructose + glucose) of nectars associated with two SNP haplotypes at *CWINV2* (*n* = 10 inbred lines per group), and **(C)** illustration of the range of floret sizes in cultivated sunflowers and the effect of decreasing floret size (from start to end of arrows) on visitation by wild bees as observed by Portlas et al. (2018). Significant differences between pairs in **(A,B)** indicated by differing lowercase letters.

Given the importance of floral traits to bee visitation in sunflowers and the crop’s reliance on bees, we attempted to leverage information on nectar-related traits in other plants and sunflower genetic resources to find and validate genetic markers that would enable marked-assisted breeding for improved sunflower-pollinator interactions. As a first step, we searched for sunflower homologues of *Arabidopsis thaliana* genes with known nectar-related functions (refer Table 2 from Roy et al., 2017), then examined whether variation in sunflower single nucleotide polymorhpisms (SNP) matched data on nectar volume or sugar composition from Mallinger and Prasifka (2017a). Observed phenotypic variation in inbred lines matched SNP markers from promoter or gene regions in just one case (cell wall invertase, *HaCWINV2*). When six inbred lines that varied for nectar volume and composition were grown (*n* = 4 replicates) and nectary gene expression quantified using RNA-seq, results supported the hypothesis that *HaCWINV2* governs sucrose content in sunflower nectar, as the highest sucrose line (HA 456) showed the fewest reads (**Figure 1A**). To validate the gene-trait association in sunflowers, inbred lines with unknown nectar types but SNP haplotypes matching high or low sucrose lines (*n* = 10, each group) were grown and nectar sugars determined by high-performance anion exchange chromatography, which clearly supported the role of *CWINV2* in determining sugar composition in sunflower nectar (**Figure 1B**). While sucrose may influence bee foraging in sunflowers (Waller, 1972; Pham-Delègue et al., 1994; Mommaerts et al., 2013), finding markers for another nectar-related trait, floret size, is a priority because small changes in floret size have dramatic effects on nectar access (and sunflower visitation) by wild bees (**Figure 1C**; from data of Portlas et al., 2018). Previous identification of genes that govern flower size in other plants (Krizek and Anderson, 2013) suggests that this is achievable, and analysis of a broader panel of sunflower lines has identified several quantitative trait loci (QTL) for this trait (Hulke, unpublished data). Following identification of QTL that govern nectar quality and accessibility, the next challenges are to phenotype large populations for nectar and pollen quantity and develop markers which would expedite breeding sunflowers with enhanced pollinator reward.

## Future Research Needs

Research in sunflowers and other crops demonstrates that enhancement of crop-pollinator interactions by selection on nectar-related traits is both worthwhile and feasible. In addition to demonstrating potential benefits, trade-offs and costs also should be considered. For example, because adults and larvae of many insects feed on nectar and pollen (Wäckers et al., 2007), changes intended to benefit pollinators could also impact pest management (and vice-versa; Lucas-Barbosa, 2016). Also, targeted changes to nectar-related traits could have energetic costs that limit yields, though adaptations like nectar resorption can mitigate potential costs (Nepi and Stpiczyńska, 2007). However, given the potential benefits to crops and pollinators, trade-offs or costs should not discourage development of varieties and hybrids with improved nectar or nectar-related traits, but be addressed on a case-by-case basis.

## Author Contributions

JP, CC, KF, BH, and RM conducted the studies on sunflower nectar-related traits and designed the pollination. JP, ZP, MH, RM, CC, and TP collected and analyzed the data. JP, BH, RM, and ZP planned and wrote the manuscript. All authors have read and approved the manuscript.

## Conflict of Interest Statement

The authors declare that the research was conducted in the absence of any commercial or financial relationships that could be construed as a potential conflict of interest. The handling Editor declared a shared affiliation, though no other collaboration, with one of the authors RM.
